# Experimental Study on Migration Characteristics and Profile Control Performance of Gel Foam in Fractured-Vuggy Reservoir

**DOI:** 10.3390/gels11100768

**Published:** 2025-09-24

**Authors:** Yan Xin, Binfei Li, Jingyu Zhang, Bo Wang, Aojue Liu, Zhaomin Li

**Affiliations:** 1 State Key Laboratory of Deep Oil and Gas, China University of Petroleum (East China), Qingdao 266580, China; xinyan9710@163.com (Y.X.); s23020113@s.upc.edu.cn (B.W.); liuaojue888@163.com (A.L.); 2 School of Petroleum Engineering, China University of Petroleum (East China), Qingdao 266580, China; 3 Bohai Petroleum Research Institute, Tianjin Branch of CNOOC (China) Ltd., Tianjin 300452, China; zhangjy332299@163.com; 4 School of Chemietry and Chemical Engineering, Shihezi University, Shihezi 832003, China; lizhm@upc.edu.cn

**Keywords:** fractured-vuggy reservoir, gel foam, migration characteristics, profile control performance, enhanced oil recovery mechanisms

## Abstract

Gel foam exhibits excellent applicability in fractured-vuggy reservoirs, effectively plugging flow channels and enhancing oil recovery. However, due to the harsh high-temperature environment and the complex and variable fracture-vuggy structure in reservoirs, gel foam may undergo structural changes during its migration, which can affect its flow properties and plugging efficiency. Therefore, investigating the migration characteristics of gel foam in fractured reservoirs through visual experiments is of significant practical importance. In this study, migration experiments with different foam systems were conducted using the visualized vuggy model. The migration stability of foam was characterized by combining the sweep range and liquid drainage rate, and the impact of temperature on the migration characteristics of gel foam was explored. Additionally, a profile control experiment was performed using the fractured-vuggy network model, analyzing and summarizing its mechanisms for enhancing oil recovery in fractured-vuggy reservoirs. The results showed that, in the vuggy model, compared with ordinary foam and polymer foam, gel foam showed a lower drainage rate, higher foam retention rate and wider sweep range, and could form stable plugging in fractured-vuggy reservoirs. An increased temperature accelerated the thermal expansion of gas and changes in liquid film characteristics, which led to the expansion of foam migration speed and sweep range. Although a high temperature increased the liquid drainage rate of foam, it was still lower than 3%, and the corresponding foam retention rate was higher than 97%. In addition, the gel foam had a strong profile control ability, which effectively regulated the gas migration path and improved the utilization degree of remaining oil. Compared with the first gas flooding, the recovery of subsequent gas flooding was increased by 18.85%, and the final recovery of the model reached 81.51%. Comprehensive analysis revealed that the mechanism of enhanced oil recovery by gel foam mainly included density control, foam regeneration, flow redirection, stable plugging, and deep displacement by stable gel foam. These mechanisms worked synergistically to contribute to increased recovery. The research results fully demonstrate the application advantages of gel foam in fractured-vuggy reservoirs.

## 1. Introduction

Carbonate fractured-vuggy reservoirs, as a special type of unconventional reservoir, are rich in reserves and have become one of the main fields for increasing oil reserves and production [1,2,3,4,5]. According to statistics, the fractured-vuggy reservoirs in China are large-scale ancient karst fractured-vuggy reservoirs, such as Tahe Oilfield and Lungu Oilfield situated in Tarim Basin. Crude oil is primarily distributed in large vugs, holes, and fractures, with almost no storage and permeability capacity in the matrix, making the reservoir highly heterogeneous and difficult to develop [6,7,8,9,10,11]. Therefore, when using conventional water injection development, the injected water is prone to forming channeling flow paths, leading to a reduced sweep efficiency, rapid increases in water cut, and a sharp decline in oil well production. For example, the TK765CH well group and T402 well group in Tahe Oilfield showed good water injection response characteristics at the initial stage of water injection. However, in the subsequent period, the water channeling in the injection production well group was serious, and oil production decreased [12,13]. Gas injection is also an effective method for improving recovery. By maintaining reservoir pressure and taking advantage of the density difference between gas and crude oil, it can effectively displace “roof oil” from the upper parts of the reservoir [14,15]. However, gas often preferentially flows through fractures with lower resistance, which can lead to early gas breakthrough and a cessation of increased recovery [16].

Gel foam has received widespread attention in recent years as an important method for plugging channeling and enhancing recovery in fractured-vuggy reservoirs [17,18,19]. Compared with traditional polymer foam, gel foam has outstanding performance advantages in harsh environments. The stability of polymer foam mainly depends on the viscosity of the foaming base liquid. In high-salinity and high-temperature environments, the structure and behavior of polymer chains are affected, resulting in a significant decrease in the viscosity of foam. This change accelerates the rupture and liquid separation of foam, which makes it impossible to maintain stability in a reservoir for a long time [20,21]. Gel foam can effectively overcome the limitations of polymer foam. Before gelation, the system has a low viscosity, excellent foaming ability, and strong flowability [22]. After gelation, the three-dimensional network structure formed by gel in the liquid film significantly increases the viscoelasticity of the liquid film, reduces the liquid drainage rate of the foam, and significantly enhances its thermal stability, giving it the advantages of long-term stable existence and effective plugging in high-temperature fractured-vuggy reservoirs [23,24,25,26].

Due to the harsh environment and complex structures of fractured-vuggy carbonate reservoirs, foam undergoes a complicated structural change process during migration [27,28,29,30]. Therefore, studying the migration characteristics of foam is particularly important. However, existing visual studies mainly focus on the analysis of foam migration patterns and stability at room temperature, and are primarily concentrated on ordinary foam and polymer foam systems. There is still a lack of analysis of gel foam migration patterns. The migration characteristics of ordinary foam and polymer foam in fractures or vugs are insufficient to support the understanding and derivation of gel foam migration characteristics. Therefore, it is necessary to conduct separate studies on the migration characteristics and patterns of gel foam.

In this study, based on the visualized vuggy model, the migration state and liquid drainage degree of ordinary foam, polymer foam, and gel foam were compared, and the impact of temperature on the migration pattern and sweep range of gel foam was analyzed. Then, the profile control effect of gel foam was further studied through an experiment using the fracture-vuggy network model. The novelty of this work lies in the direct visualization and quantitative comparison of the migration behavior and stability of different foams under high-temperature conditions, which has rarely been explored in previous studies. Furthermore, it provides new insights into the profile control and enhanced oil recovery mechanisms of gel foam. We hope that the results obtained from this study will provide a theoretical basis and reference for the application advantages of gel foam in harsh fractured-vuggy reservoirs.

## 2. Results and Discussion

### 2.1. Study on Migration Behaviors in Vugs

Based on the visualized vuggy model, the migration behavior of foam in vugs was studied. The migration state and liquid drainage degree of ordinary foam, polymer foam, and gel foam were compared, and the influence of temperature on the migration behavior and sweep range of gel foam was analyzed.

#### 2.1.1. Comparative Analysis of Migration Behaviors of Different Foam Systems in Vugs

The model was placed vertically in an oven at 60 °C, and the migration behavior and filling ability of ordinary foam, polymer foam, and gel foam in vugs were compared and studied. During the migration of foam in the vugs, due to the dynamic stability of foam being weaker than its static stability, liquid drainage generally occurred. The foam’s drained liquid was transparent and slightly different from the model, which was not conducive to observation. Therefore, Photoshop was used to extract and color (sky blue) the liquid in images for better visualization.

**Figure 1:**
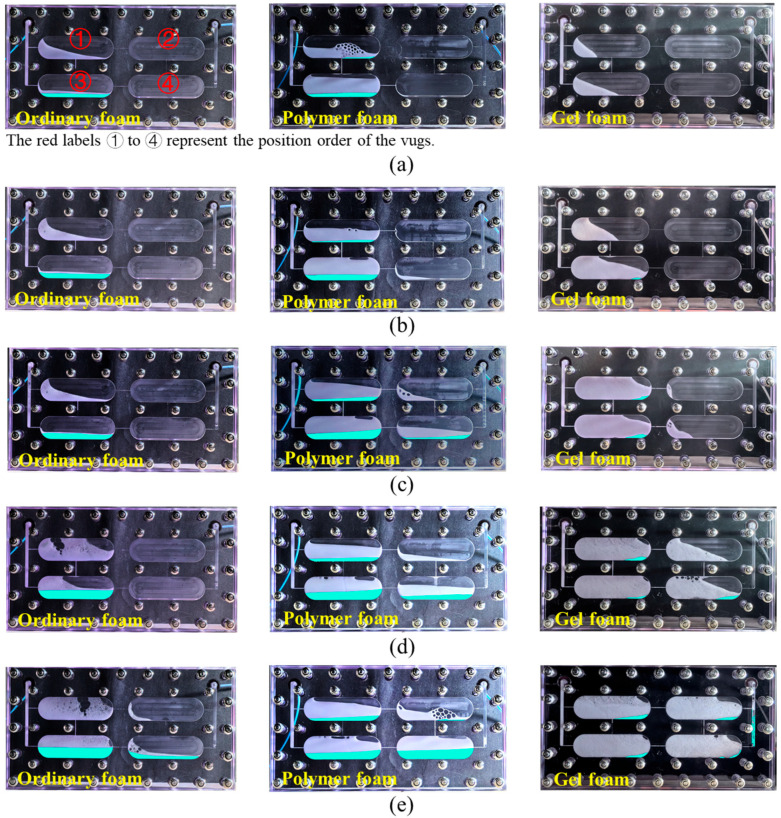
Migration state and sweep range of foam in the visualized vuggy model: (**a**) 0.2 PV; (**b**) 0.4 PV; (**c**) 0.6 PV; (**d**) 0.8 PV; and (**e**) 1.0 PV.

An experimental image of the migration state and sweep range of different foams in vugs, changing with the injection volume, is shown in Figure 1. It is not difficult to find that when the injection volume was 0.8 PV, the sweep range of ordinary foam to vugs was only limited to vugs ① and ③ (near-well zone), and when the injection volume exceeded 0.8 PV, the foam entered vugs ② and ④ (far-well zone). Compared to ordinary foam, polymer foam exhibited a significant improvement in sweep range under the same injection volume. As shown in Figure 1b, polymer foam began to enter vugs ② and vug ④ when 0.4 PV was injected, and the sweep range was further expanded with a further increase in injection volume. For gel foam, although it started to enter vugs ② and ④ at an injection volume of 0.4–0.6 PV (higher than polymer foam but lower than ordinary foam), it occupied a larger sweep range in the subsequent injection process (0.6–1 PV).

**Figure 2:**
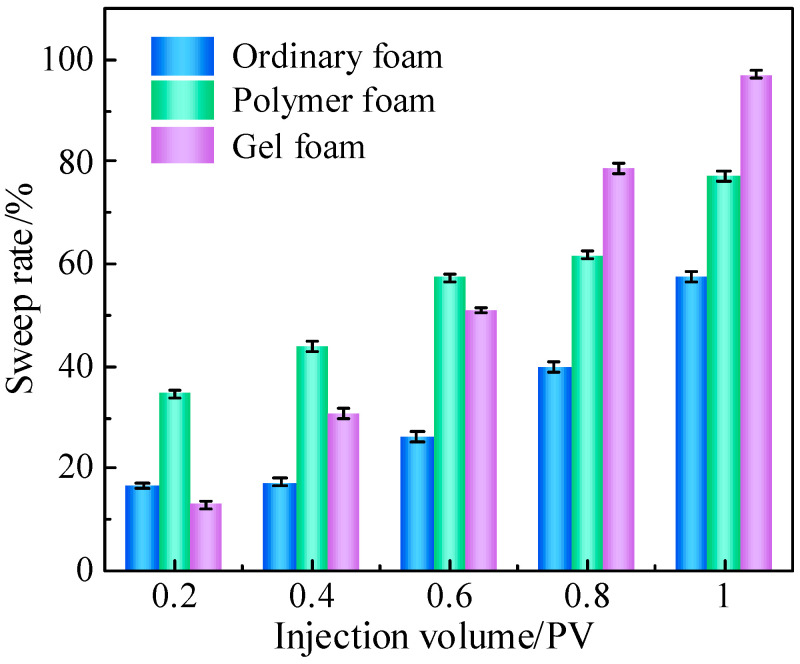
Sweep rate of foam at different injection volumes.

To obtain more accurate data for further analysis, the sweep rate, liquid drainage rate, and foam retention rate of each foam system were calculated using the image recognition software ImageJ 1.53, tracking changes with varying injection volumes. The sweep rate was defined as the ratio of the sweep volume of the foam system to the model volume. The variations in the sweep rate of the three foam systems are shown in Figure 2, and an increase in injection volume led to an expansion of the sweep rate. Different foam systems exhibited different sweep range growth rates, which were mainly affected by the viscosity of the system. Ordinary foam had a low viscosity and fast migration speed. However, due to its poor stability, the foam was easy to rupture, resulting in a sweep rate of only 16.59% at 0.2 PV. Even at 0.4 PV, the sweep rate was only 17.34%, and the final rate was only 57.53%. Polymer foam had a moderate viscosity and medium migration rate, but its stability was higher than that of ordinary foam. When 0.2 PV was injected, the sweep rate was as high as 34.55%. In the early injection stage (0–0.6 PV), it demonstrated an excellent sweeping effect. Limited by the stability in the later stage of injection, the rate of increase in sweep range slowed down, with the final sweep rate reaching 77.09%. Compared with the other two kinds of foam systems, gel foam had a high viscosity, resulting in a slower migration rate, and its sweeping ability was not outstanding in the early stage of injection, with a sweep rate of only 50.91% at 0.6 PV. However, in the later stage of injection, due to its high foam stability, the sweep rate of gel foam increased rapidly, eventually reaching 97.10%.

During the migration process of the three foam systems in the vugs, different liquid drainage behaviors were also observed (represented by the blue areas in the images). In Figure 1, it can clearly be seen that the drained liquid volume of gel foam was much lower than that of ordinary foam and polymer foam, which is consistent with the foam’s static stability. In fractured-vuggy reservoirs, the gravitational segregation effect is significant. As shown in Figure 1, ordinary foam and polymer foam experienced rapid liquid drainage, with the drained liquid converging through fractures to the bottom of the vugs, while the gas diffused to the top of the vugs, forming a gas–liquid separation state. From top to bottom of the vuggy model, the layers consisted of gas, high-dryness foam, wet foam, and drained liquid. The gas at the top and the drained liquid (containing surfactant) at the bottom did not have the opportunity to mix and generate foam again. In contrast, gel foam only discharged a small amount of liquid during migration, with the drained liquid accumulating in the lower right corner of each vug (the blue areas).

**Figure 3:**
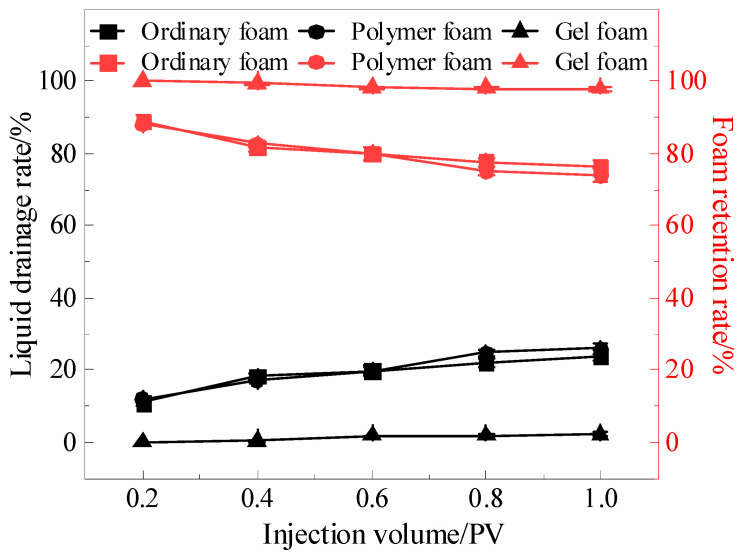
Liquid drainage rate and foam retention rate of different foams during migration.

To quantify the stability of different foam systems, the liquid drainage rate was defined as the ratio of the drained liquid volume to the sweep volume, and the foam retention rate was defined as the ratio of the foam volume to the sweep volume. The changes in the liquid drainage rate and foam retention rate with varying injection volumes are shown in Figure 3. As the injection volume increased, the liquid drainage rate gradually increased, while the foam retention rate gradually decreased. Among the foam systems, the liquid drainage rate and foam retention rate of ordinary foam and polymer foam exhibited larger fluctuations. When the injection volume increased from 0.2 PV to 1 PV, the drainage rate of ordinary foam rose from 11.03% to 23.89% (with the corresponding foam retention rate decreasing from 88.97% to 76.11%), while the drainage rate of polymer foam increased from 11.88% to 25.99% (with the corresponding foam retention rate decreasing from 88.12% to 74.01%). Generally speaking, polymer foam has a higher stability than ordinary foam, but in this experiment, the liquid drainage rates of the two systems were quite similar. The analysis suggests that although ordinary foam has a lower stability and faster liquid drainage, the dryness of the residual foam at the top of the vugs was higher, allowing it to strongly adhere to the vug walls and thereby maintain a more stable foam structure, increasing the foam retention rate. However, the changes in the drainage rate and foam retention rate of gel foam were much smaller. Before and after injection, the drainage rate of gel foam increased from 0% to 2.15% (with the corresponding foam retention rate decreasing from 100% to 97.85%).

#### 2.1.2. Effect of Temperature on the Migration Behavior of Gel Foam

The model was vertically placed in an oven with temperatures set sequentially at 30 °C, 60 °C, and 100 °C to compare and study the effects of temperature on the migration behavior, liquid drainage rate, and sweep range of gel foam in the vugs. The image processing method was the same as in Section 2.1.1.

**Figure 4:**
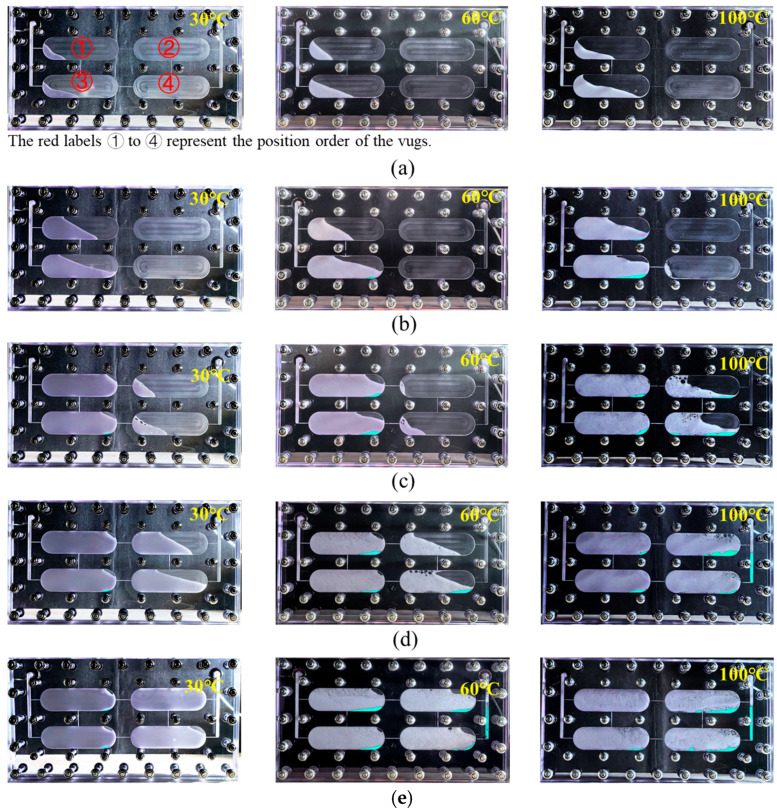
Influence of temperature on the migration and sweep range of gel foam in the visualized vuggy model: (**a**) 0.2 PV; (**b**) 0.4 PV; (**c**) 0.6 PV; (**d**) 0.8 PV; and (**e**) 1.0 PV.

Experimental images showing the migration state and sweep range of gel foam in the vuggy model at different temperatures, with varying injection volumes, are shown in Figure 4. At 30 °C and 60 °C, gel foam first entered vugs ① and ③, and at injection volumes of 0.4–0.6 PV, it began to enter vugs ② and ④. When the temperature reached 100 °C, gel foam entered vug ④ at 0.4 PV, which was believed to be caused by foam expansion due to the high temperature. However, after injecting 1 PV, gel foam was able to sweep most of the vugs in all temperature conditions.

**Figure 5:**
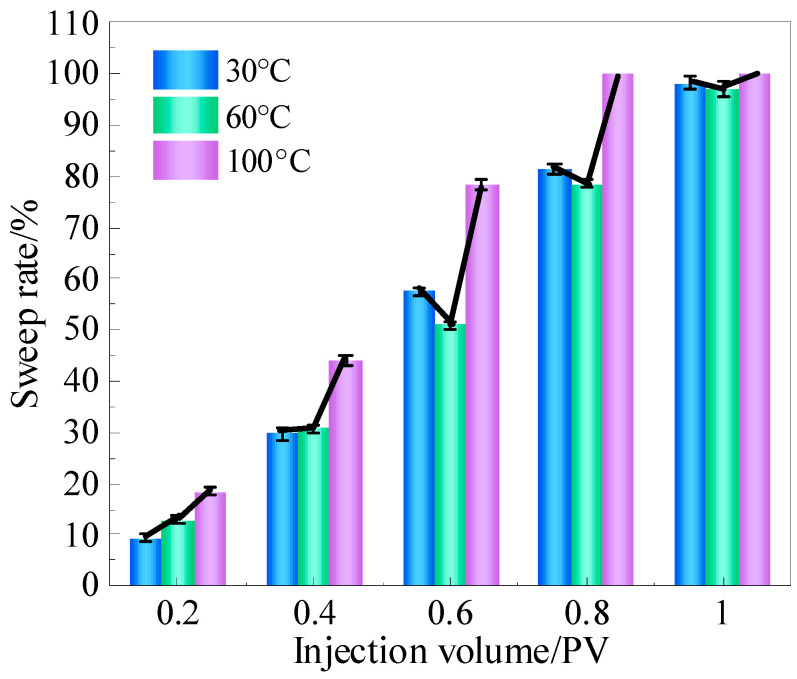
Influence of temperature on the sweep rate of gel foam.

The specific sweep rate of gel foam is shown in Figure 5. The impact of temperature on the sweep rate of gel foam did not follow a simple trend at different injection stages. The migration and sweep range of the gel foam system in high-temperature vugs were mainly influenced by a combination of factors, including the gelation characteristics of the liquid film, gas thermal expansion, and migration speed. In the early injection stage (0–0.4 PV), as the temperature increased, the sweep rate of gel foam gradually increased. At experimental temperatures, although the strength of the gel film increased with temperature, unlike a pure gel, the thermal expansion of the gas in the gel foam system became more prominent. The gas expansion not only increased the foam volume to some extent, thus expanding the sweep range, but also led to thinning of the liquid film, reducing the system’s viscosity and further increasing the flow velocity, which enabled the foam to occupy more reservoir space quickly. Additionally, the reduced viscoelasticity of the liquid film due to the higher temperature and weakened adsorption force on the vug wall further exacerbated this trend. In the later injection stage (0.4–1 PV), as the temperature increased, the sweep rate of gel foam showed a trend of first decreasing and then increasing. With the long-term retention and significant accumulation of foam in the vugs, the expansion of the gas was partially suppressed, which enhanced the adsorption capacity of gel foam to the vug walls, particularly at 60 °C. At 30 °C, the gel foam’s liquid film strength was low, the foam system had a low viscosity, and the migration speed was fast, which resulted in an increased sweep range. However, at 60 °C, the gas expansion effect weakened, the system’s viscosity gradually recovered, and the liquid film strength was higher, resulting in a slower migration speed and a deceleration in the growth rate of the sweep range, ultimately leading to a sweep rate lower than that at 30 °C. At the high temperature of 100 °C, the foam was still mainly influenced by gas expansion and the migration speed was fast, resulting in a greater sweep rate.

**Figure 6:**
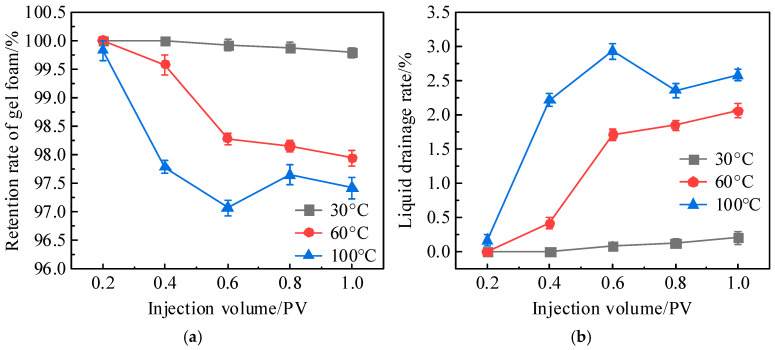
Effect of temperature on liquid drainage rate and retention rate of gel foam: (**a**) retention rate of gel foam and (**b**) liquid drainage rate.

As the temperature increased, gel foam experienced varying degrees of liquid drainage, which accumulated at the bottom of the vug due to gravity, as shown by the blue areas in Figure 4. The liquid drainage rate and foam retention rate of gel foam at different temperatures were calculated and are shown in Figure 6. As shown in Figure 6, with an increase in temperature, the liquid drainage rate of gel foam gradually increased, while the foam retention rate gradually decreased. This was primarily due to foam breakdown and liquid film separation caused by a high temperature. However, within the temperature range of 30–100 °C, the liquid drainage rate of gel foam remained below 3% and the foam retention rate stayed above 97%. Overall, temperatures within this range had little impact on the liquid drainage rate and foam retention rate of gel foam. This was largely due to the three-dimensional network structure of the liquid film formed by gel foam, which provided it with a strong stability and shear resistance. This structure resisted thermal effects, preventing significant liquid drainage even in moderately high-temperature environments [31,32,33,34]. Additionally, the network structure helped to extend the effective duration of gel foam in the vugs, ensuring that it could maintain its performance over a longer period.

In addition, by comparing the experimental results in Section 2.1.1, it can be seen that the negative influence of temperature on foam migration behavior also changed significantly with the composition of the foam system. With its high stability, gel foam had a strong adaptability to fractured-vuggy reservoirs, which need deep profile control and long-term plugging. In contrast, at 60 °C, the thermal stability of ordinary foam was already poor, with rapid foam rupture, a small scanning range, and severe liquid separation. Polymer foam exhibited a certain heat resistance due to its viscosity-increasing and foam-stabilizing effects. However, its viscosity still decreased over time and its stability was relatively poor. When the temperature continues to rise, the stability of these two types of foams will further significantly decrease, resulting in poor application effects, so they are not suitable for high-temperature reservoir environments [35,36].

**Figure 7:**
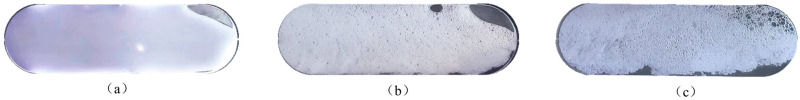
Effect of temperature on the morphology characteristics of gel foam in vug (1 PV): (**a**) 30 °C; (**b**) 60 °C; and (**c**) 100 °C.

Figure 7 shows the effect of temperature on the foam morphology as gel foam migrated through vug ④, with an injection volume of 1 PV. It is visually evident that at a lower temperature, the foam had a thick liquid film, with small and uniform bubbles, resulting in a dense foam system in the vug with no noticeable liquid drainage. However, as the temperature increased, the liquid film gradually became thinner and the foam became sparser. Small bubbles continuously coalesced into larger bubbles, as shown in the upper-right corner of Figure 7c. At the same time, due to gravity, the vug displayed a distribution from top to bottom, consisting of high-dryness foam, wet foam, and drained liquid.

### 2.2. Profile Control Performance and Enhanced Oil Recovery Mechanism of Gel Foam in Fractured-Vuggy Network Model

#### 2.2.1. Profile Control Characteristic of Gel Foam

**Figure 8:**
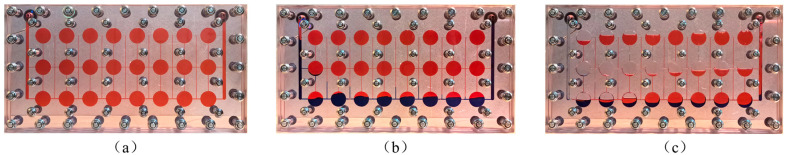
Remaining oil distribution in fractured-vuggy model before injecting gel foam: (**a**) initial saturated oil state; (**b**) after waterflooding; and (**c**) after gas flooding. Color codes: Oil (red), Water (blue), Gas (colorless), Foam (white).

In the figure, the red areas represent simulated oil, the blue areas represent injected water, the colorless areas represent gas, and the white area represents foam. Figure 8 illustrates the distribution of remaining oil in the fractured-vuggy network model after different displacement processes, as follows: the initial saturated oil state, waterflooding, and gas flooding. Due to the vertical placement of the model and the influence of gravity and density differences, the injected water moved along the bottom of the model, preferentially sweeping the oil at the bottom. The low flow resistance caused by the 1.2 mm wide horizontal fracture at the bottom also aided in the advancement of the water. Once the water reached the production well, this indicated that a channeling flow path had formed, and no more oil was produced. In contrast, due to the lower density of gas, it mainly swept the oil in the upper part of the model. The sweep range of gas was constrained by the opening of the horizontal fractures and was limited to the middle vugs. After the gas flooding process was completed, the remaining oil was primarily concentrated in the far-well zone and at the bottom of the vugs.

**Figure 9:**
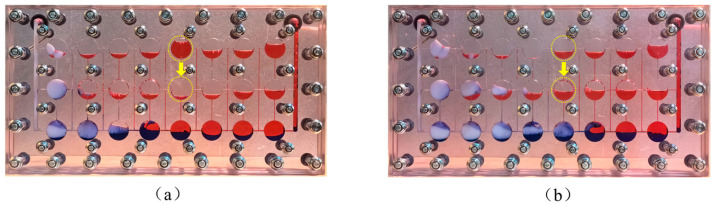
Migration state of gel foam in fractured-vuggy model: (**a**) 0.2 PV and (**b**) 0.4 PV. Color codes: Oil (red), Water (blue), Gas (colorless), Foam (white).

After gas flooding, 0.4 PV of gel foam was injected to plug the channeling flow paths. The migration state and plugging range of the gel foam in the fractured-vuggy network model are shown in Figure 9. Unlike water and gas, gel foam is composed of a gel system and gas system, with a density between that of water and gas. This gives it strong profile control and density regulation capabilities. Gel foam preferentially entered the bottom vugs along large fractures. As the foam accumulated, the flow resistance increased, causing the foam to divert into smaller fractures, gradually expanding its sweep volume. In the vertical direction, the gel foam could almost reach the upper, middle, and lower vug spaces, with the first row of vugs near the well nearly completely filled with it. In the horizontal direction, the foam displayed a step-like distribution from top to bottom. Throughout the process, only a small amount of crude oil was recovered. Because only 0.4 PV of gel foam was injected, it primarily affected the near-well zone. Consequently, the remaining oil in the far-well zone, under the influence of gravity, moved through fractures to the bottom vugs, leading to a redistribution of the remaining oil in the vugs, as indicated by the yellow markers in Figure 9.

**Figure 10:**
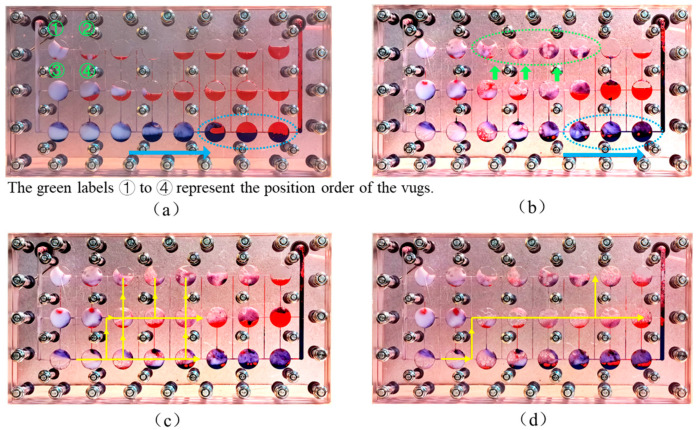
Fluid distribution image in the fractured-vuggy model under different injection volumes during subsequent gas flooding stage: (**a**) 0 PV; (**b**) 0.2 PV; (**c**) 0.3 PV; and (**d**) 0.5 PV. Color codes: Oil (red), Water (blue), Gas (colorless), Foam (white).

**Figure 11:**
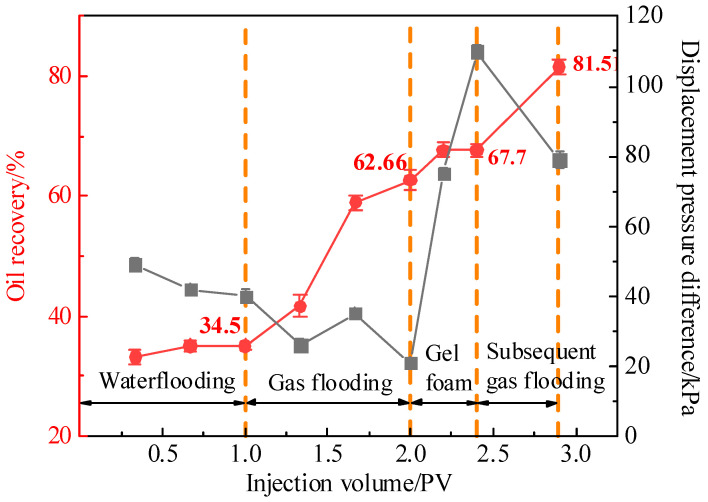
The oil recovery and displacement pressure difference at different stages.

During the subsequent gas flooding stage, the fluid migration paths and distribution in the fractured-vuggy model became more complex. To make the oil, water, gas, and foam more visible, Photoshop was used to adjust the color of the images, as shown in Figure 10. From the figure, it is visually evident that after the subsequent gas flooding, the gas migration path changed and the remaining oil decreased significantly, indicating that more crude oil was being recovered. This observation could be related to the change in pressure in the model. The increase in pressure in the subsequent gas flooding process forced the gas to change the migration path and then pushed more oil to flow to the production well, resulting in a decrease in remaining oil saturation and the enhancement of oil recovery. As shown in Figure 11, the displacement pressure difference in the subsequent gas flooding stage was 3.8 times that in the initial gas flooding stage, the stage oil recovery rate reached 13.81%, and the final recovery rate of the model was 81.51%. The correlation between pressure, oil recovery data, and migration path is helpful to strengthen the understanding of the oil recovery enhancement mechanism in the subsequent gas flooding stage.

#### 2.2.2. Mechanism Analysis of Enhancing Oil Recovery by Gel Foam

The enhanced oil recovery results of the profile control experiment can be analyzed from the following two aspects: the migration paths of displaced fluids and the distribution of remaining oil in the model. The mechanism of enhanced gel foam recovery can be summarized in the following five points:

(1) Density regulation

The density of foam lies between that of gas and liquid phases, allowing it to enter regions that are inaccessible to both gas and liquid. Under density regulation, foam can sweep the remaining oil in the middle regions of the reservoir.

(2) Foam regeneration

On one hand, gel foam has a higher liquid film viscosity compared to ordinary foam, making it more difficult to foam at the same gas-to-liquid ratio, which results in a lower foam volume. However, this also means that it has strong potential for secondary foam generation later on. On the other hand, gel foam has a thick liquid film, high viscoelasticity, and good extensibility, allowing it to effectively encapsulate gas during the gas expansion process without breaking. As shown in Figure 10, with an increase in the injected gas volume, large areas of regenerated foam were observed, where some of the gas existed in the form of foam. Compared to gas flooding, foam flooding had a stronger oil-washing efficiency and a larger sweep volume, enabling more effective recovery of the remaining oil. Notably, regenerated foam often has larger bubble sizes and thinner liquid films, which help reduce the viscosity of the foam system, increase the foam migration speed, and expand the sweep volume. Although bubbles with thin liquid films are more prone to breakage and have a sparser distribution, continuous gas supply that leads to sustained foam regeneration compensates for this limitation.

(3) Flow redirection

As shown in Figure 8c, before the foam injection, the injected gas eventually migrated along the central vugs, with the flow path primarily concentrated in the horizontal direction. During subsequent gas flooding, as shown by the green circle and arrows in Figure 10b, this section of the vugs, which previously contained only a small amount of remaining oil, as shown in Figure 10a, was filled with foam, and the remaining oil was recovered. This indicates that under the plugging effect of gel foam, gas is able to open up a vertical migration path, carry liquid upward, and sweep the top vugs.

During the foam regeneration process, the pressure field within the entire fractured-vuggy model changed, causing the gas flow path to shift accordingly. When approximately 0.3 PV of gas was injected, the gas flowed along the yellow arrow shown in Figure 10a. Due to the distribution of regenerated foam in various spaces, especially the accumulation of foam in the upper vugs, the vertical expansion path of the gas was plugged. As a result, when 0.5 PV was injected, the gas flow path significantly decreased, and the vertical flow path shifted to the far-well zone. The displacement pressure difference in the subsequent gas flooding stage was 3.8 times that in the initial gas flooding stage, which subsequently promoted the recovery of remaining oil in the far-well zone, so the recovery of the subsequent gas flooding could be increased by 13.81%.

(4) Stable plugging

As shown in Figure 10, the gel foam in vugs ①, ③, and ④ remained stable after the subsequent gas flooding process. This stability is primarily attributed to the high stability and high oil resistance provided by the gel shell.

(5) Deep displacement by stable gel foam

During subsequent gas injection, part of the gel foam underwent secondary foaming due to shear forces from the gas, forming new bubbles. Another part of the gel foam remained stably over a long period. Under the influence of gas and regenerated foam, this stable gel foam continued to migrate deeper and displace more crude oil. As shown in Figure 10a,b, the foam in vug ④ moved toward vug ②, demonstrating the vertical migration process of stable gel foam. The blue circles and blue arrows show the horizontal migration process of stable gel foam. Even after injecting 0.5 PV of gas, the foam maintained its structural integrity.

These five mechanisms work in coordination and promote each other, achieving the goal of “plugging and enhancing recovery”.

### 2.3. Comparison with Previous Studies

In order to better understand the improvement brought about by our work, we compared our work with previous work considering the aspects of foam type, experimental temperature range, and main findings, as shown in Table 1. The improvement of our work is effectively reflected through clear comparison, which mainly includes the following three aspects: (1) the migration behavior differences of three different foam systems were compared; (2) the migration behavior of gel foam in a wider temperature range (30–100 °C) was studied; and (3) a more detailed and comprehensive mechanism of gel foam to enhance oil recovery was put forward and analyzed.

## 3. Conclusions

Experiments on foam migration behavior and profile control capabilities were conducted. The conclusions are as follows:

(1) Compared to ordinary foam and polymer foam, gel foam exhibited a lower liquid drainage rate, higher foam retention rate, larger sweep range, and stronger reservoir adaptability.
(2) An increased temperature accelerated the thermal expansion of gas and changes in liquid film characteristics, which enhanced the migration speed and sweep range of gel foam. Within the temperature range of 30–100 °C, the drainage rate remained below 3%, the corresponding foam retention rate was above 97%.
(3) Gel foam demonstrated a strong residual oil mobilization capacity. The displacement pressure difference in the subsequent gas flooding stage was 3.8 times that in the initial gas flooding stage, the oil recovery was 18.85% higher than that in the initial gas flooding stage, and the final recovery of the model reached 81.51%.
(4) The relationship between displacement pressure difference, oil recovery data, and migration path was helpful to understand the mechanisms underlying enhanced gel foam oil recovery. The mechanisms include density regulation to sweep the middle of the reservoir, gas shear promoting foam regeneration, flow redirection to alter the gas migration path and expand the gas sweep range, maintaining long-term plugging, and gas and regenerated foam pushing stable gel foam deeper into the reservoir. These mechanisms work synergistically to achieve efficient plugging and enhance the recovery of residual oil.

The current study was conducted using visualized models, which may not fully replicate the complexities of real reservoirs. Future work should incorporate larger-scale models or field-scale studies to better validate the observed effects and refine the recovery mechanisms.

## 4. Materials and Methods

### 4.1. Materials

**Figure 12:**
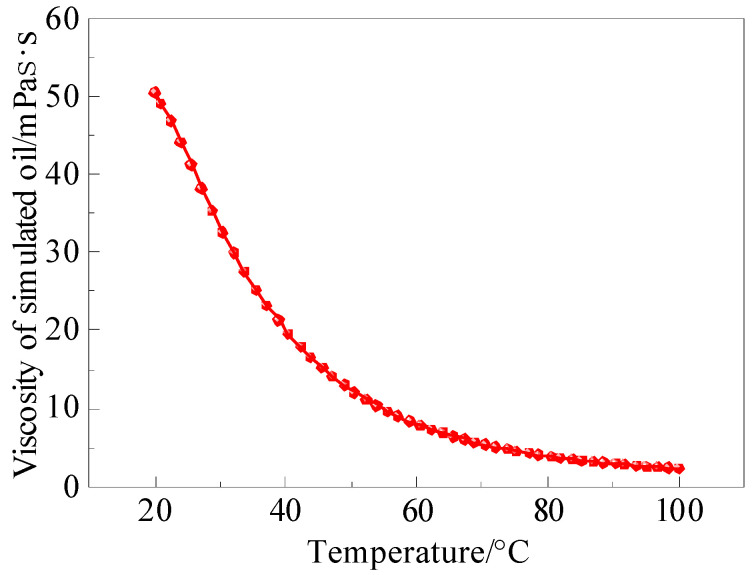
Viscosity–temperature curve of simulated oil.

Cellulose-based polymer HPG-X (Renqiu Gaoke Chemical Materials Co., Ltd., Renqiu, China). Crosslinking agent TC-1 (China Tianchen Engineering Co., Ltd., Tianjin, China). Water-soluble foaming agent (Jiangsu Suolong Fire Technology Co., Ltd., Yangzhou, China). N_2_ (purity 99.9%, Qingdao Tianyuan Gas Manufacturing Co., Ltd., Qingdao, China). Deionized water (resistivity of 18.0 MΩ·cm) was prepared using an ultrapure water instrument in the laboratory. Anti-wear hydraulic oil (Dongguan Kelasuo Lubricating Oil Co., Ltd., Dongguan, China) was used as the simulated oil for the experiment. The viscosity–temperature curve of the simulated oil was measured using an Anton Paar rheometer, and the test result is shown in Figure 12. To facilitate observation, oil red was used to dye the simulated oil, and brilliant blue was used to dye the water, distinguishing the distribution of different fluids in the model. Both oil red and brilliant blue were purchased from Macklin Biochemical Technology Co., Ltd. (Shanghai, China).

Preparation of gel foam base solution: First, a certain proportion of HPG-X was added to deionized water and stirred at 1500 r/min for 8 h to prepare the polymer solution. The foaming agent and crosslinking agent were then sequentially added to the polymer solution and stirred at 300 r/min for 10 min to obtain a uniform composite foaming system base solution. The base solution was left to stand for a period of time to allow gelation to occur.

### 4.2. Experimental Setup

ISCO pump (American Teledyne Co., Ltd., Lincoln, NE, USA). Core holder (Hai’an Huacheng Scientific Instruments Co., Ltd., Haian, China), Constant-temperature magnetic stirrer (Model: HMS-901D, Shenzhen Boda Jingke Biotechnology Co., Ltd., Shenzhen, China). Electronic balance (Model: JJ224BC, Changshu Shuangjie Testing Instruments Factory, Changshu, China), and other supporting equipment, including a high-temperature oven, intermediate container, one-way valve, foam generator, camera, glass rod, graduated cylinder, beaker, stopwatch, etc.

**Figure 13:**
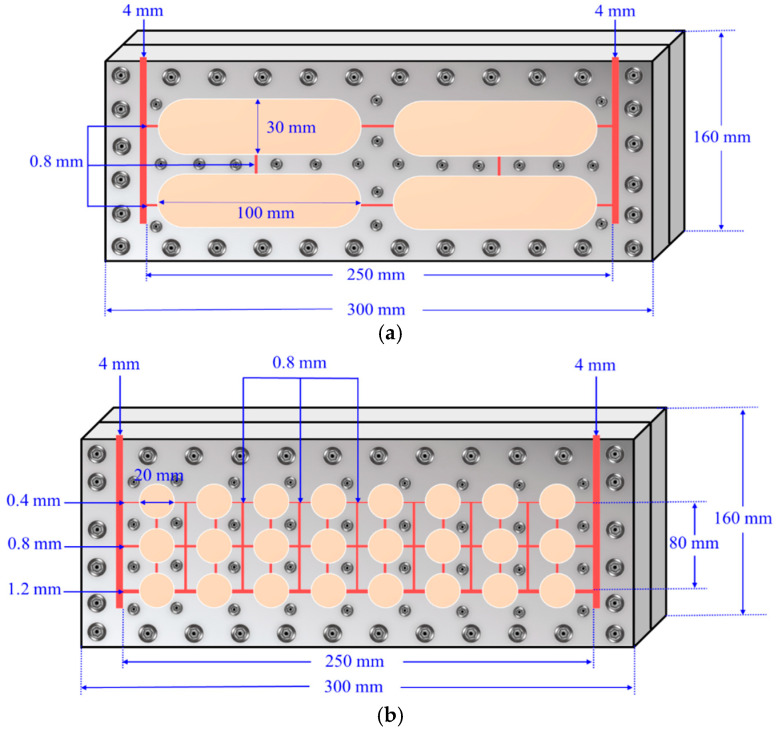
Schematic of the visualized model: (**a**) vuggy model and (**b**) fractured-vuggy network model.

To realistically simulate the fluid migration and distribution characteristics, a visualized vuggy model and fractured-vuggy model were designed and fabricated based on the principle of geometric similarity, and the physical properties and injection parameters of the fluid were designed through motion similarity and dynamic similarity, thus ensuring the similarity of the fluid flow in the model and the actual reservoir. The specific design method has been introduced in detail in the existing literature [34]. The models were made from Pasmo high-performance special transparent material, which features optical transparency, an ultra-high strength, resistance to high temperatures and pressures, ease of processing, an excellent dimensional stability, and superior physical and mechanical properties. The material can withstand temperatures up to 120 °C under normal pressure. The models are as follows: (1) Visualized vuggy model: The total length of the model is 300 mm, and the height is 160 mm. Two vertical fractures, each 4 mm wide, are designed as the injection and extraction ends. The interior of the model features four regularly arranged vugs, each 100 mm long and 30 mm high. These vugs are connected by fractures 0.8 mm wide, which link the vugs to each other, as well as to the injection and extraction ends. The depth of all fractures and vugs is 4 mm. The internal volume of the model is 49.032 mL, and the overall design is shown in Figure 13a, with the panels fastened together by screws. (2) Visualized fractured-vuggy network model: The total length of the model is also 300 mm, with a height of 160 mm. Similar to the vuggy model, two vertical fractures of 4 mm width are designed as the injection and extraction ends. Inside the model, 24 regularly arranged circular grooves are designed, each with a diameter of 20 mm. The vugs are connected by fractures, with vertical fractures having a width of 0.8 mm and a height of 80 mm, and horizontal fractures varying in length and width, around 250 mm long, with widths decreasing from 1.2 mm at the top to 0.4 mm at the bottom. All fractures and vugs have a depth of 4 mm, and the internal volume of the model is 37.664 mL. The overall design is shown in Figure 13b, with the panels fastened together by screws.

### 4.3. Migration Experiment of Foam in Vuggy Model

**Figure 14:**
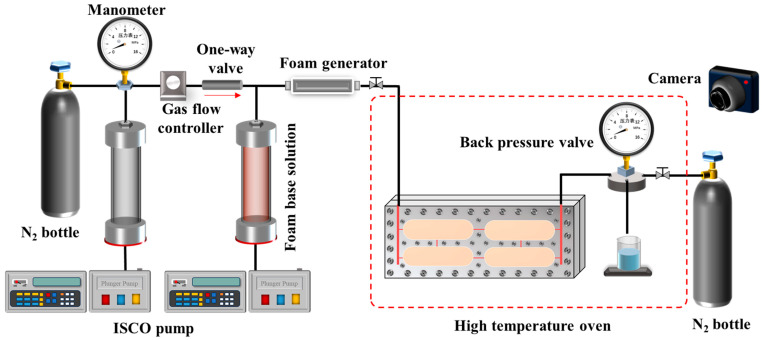
Schematic of the experimental setup of migration of foam in vugs.

The migration experiment studied the migration behavior and sweep range of foam in vugs under different influencing factors. The specific experimental steps were as follows:

(1) The foaming base solution was put into the intermediate container. N_2_ was filled into the intermediate container and adjusted in real time, with the pressure kept at about 0.5 MPa. The pressure of the back pressure valve was set at 0.5 MPa. The experimental devices were connected according to Figure 14, and the camera was turned on to record the experimental process.
(2) The gas–liquid volume ratio was maintained at 2:1, with N_2_ injected at a rate of 0.66 mL/min and the foaming solution injected at a rate of 0.33 mL/min. Foam was generated as the gas and solution passed through the foam generator. Once the foam output stabilized, it was connected to the experimental model. The total foam injection volume was 1 PV.
(3) The experimental model was cleaned, the foam system was replaced, or the temperature was changed according to the experimental parameters shown in Table 2. Steps (1) and (2) were repeated until the experiment was completed.

The composition of the three foam systems were as follows: Ordinary foam (0.8 wt% foaming agent). Polymer foam (0.3 wt% HPG-X + 0.8 wt% foaming agent). Gel foam (0.3 wt% HPG-X + 0.2 wt% TC-1 + 0.8 wt% foaming agent). Due to the temperature tolerance of the visual model, the high-temperature conditions in this study were limited to 120 °C. Therefore, experiments were conducted at 30 °C, 60 °C, and 100 °C. The controlled variable method was used, meaning that only one temperature was set in the analysis of the effects of the foam system on migration behavior. Considering that ordinary foam and polymer foam struggle to maintain stable forms at 100 °C, leading to unclear experimental results, the final temperature chosen for the experiments was 60 °C.

### 4.4. Profile Control Experiment of Gel Foam in Fractured-Vuggy Network Model

Fractured-vuggy reservoirs tend to develop channeling flow during extraction, resulting in low oil recovery. To study the profile control effect of gel foam and the mechanism for improving recovery, oil displacement experiments with the visualized fractured-vuggy network model were conducted. The experimental steps were as follows:

(1) The gel foam base solution was placed in the intermediate container, N_2_ was charged into the container, and the pressure was maintained at around 0.5 MPa. The back pressure valve pressure was set to 0.5 MPa. The visual experimental model (Figure 13b) was saturated with simulated oil, and the experimental pipelines were connected according to Figure 14. The model was kept vertically at all times, and the camera was turned on to record the experimental process.
(2) Water was directly injected into the model using an ISCO pump, with injection rate of 1 mL/min and injection volume of 1 PV. The oil production volume and displacement pressure difference were recorded.
(3) After waterflooding, gas was injected into the model at a speed of 1 mL/min, and the injection volume was 1 PV. The corresponding oil production volume was recorded.
(4) The gas–liquid ratio was maintained at 2:1, with N_2_ injected at a rate of 0.5 mL/min and the gel foam base solution injected at a rate of 1 mL/min. Foam was generated when the gas and solution passed through the foam generator. Once the foam output stabilized, it was connected to the experimental setup, with a foam injection volume of 0.4 PV. The oil production volume and displacement pressure difference were recorded.
(5) After foam injection, gas continued to be injected into the model at a rate of 1 mL/min, with an injection volume of 0.5 PV. The oil production volume and displacement pressure difference were recorded.

## Figures and Tables

**Table 1:** Summary of key findings from current and previous studies.

Study	Foam Type	Experimental Temperature Range	Main Findings
Liang et al. [37]	Ordinary foam	20 °C	Dynamic flow behavior and particle size change mechanism of foam in micro fracture-vuggy structures
Xu et al. [38]	Ordinary foam	60 °C	Static distribution and coalescence characteristics of foam in the fractures and the cavern of the microetched model
Li et al. [39]	Ordinary foam	20 °C	Foam generation, propagation and mobility characteristics in visualized fracture models. in fractures
Yang et al. [40]	Polymer foam	25 °C	Flow behavior of foam-assisted nitrogen flooding in two-dimensional visual fracture-vuggy model and main EOR mechanism
Wen et al. [41]	Gel foam	20 °C	Flow behavior of foam in visible fracture-vuggy model from one-dimensional to three-dimensional
Current work	Ordinary foam Polymer foam Gel foam	30–100 °C	The difference of foam migration characteristics in vugs under the influence of different foam types and temperatures, EOR mechanism of gel foam in fractured-vuggy reservoirs

**Table 2:** Parameter of visualized vuggy experimental model.

Foam System	Temperature/°C	Injection Rate/(mL/min)	Injection Volume/PV
Ordinary foam	60	1	1
Polymer foam			
Gel foam			
Gel foam	30	1	1
	60		
	100		

## Data Availability

The data presented in this study are available on request from the corresponding author.
